# The Importance of Imaging in the Detection of Intraoral Foreign Body

**DOI:** 10.7759/cureus.39500

**Published:** 2023-05-25

**Authors:** Sidesh Partheeban, Adrian Chan, Jason Diljohn, Nicolette Cassim, Fidel S Rampersad

**Affiliations:** 1 Department of Radiology, San Fernando Teaching Hospital, San Fernando, TTO; 2 Department of Radiology, The University of the West Indies, St. Augustine, TTO

**Keywords:** computed tomography (ct) imaging, neuroradiology, neck imaging, radiology, head and neck

## Abstract

Intraoral foreign bodies (IOFBs) can be seen incidentally on computed tomography (CT) imaging and may mimic pathology. It is therefore important to identify the imaging features of a comestible intraoral foreign body and differentiate them from true pathology to avoid unwarranted patient distress and further imaging or procedures that are unnecessary and costly.

This case describes a 31-year-old male who presented to the emergency room following a fall from an eight-foot height, with loss of consciousness for five minutes and right periorbital edema. Subsequent CT imaging of the facial bones revealed multiple facial and orbital fractures as well as a circumscribed, ovoid, hyperdensity with internal air pockets within the inferior left buccal space, which was diagnosed as an intraoral foreign body. Here, we aim to highlight the imaging features of this particular case of comestible intraoral foreign body.

## Introduction

Intraoral foreign bodies (IOFBs) can be seen on imaging performed in both the outpatient and emergency department settings. While it is common practice for patients to be asked to remove any foreign body from their oral cavity prior to imaging, occasionally, this may not happen, especially in emergency settings with a large number of patients and rapid clinical assessments. This potential pitfall can result in IOFB being reported as pathological lesions, such as traumatic foreign bodies, mucosal neoplasms, or soft tissue abscesses, by an unaware radiologist [1,2]. Current literature on the radiological findings of comestible IOFBs is relatively scarce. Therefore, this case aims to review the computed tomography (CT) imaging features of a comestible IOFB to reduce the ambiguity of these incidental findings and avoid undue distress to patients and unnecessary additional procedures due to incorrect diagnoses [2].

## Case presentation

A 31-year-old male presented to the emergency room following a fall from an eight-foot height at his workplace, with loss of consciousness for a duration of five minutes, and no associated vomiting or seizure activity. Clinical examination showed right periorbital edema with normal eye movements, no diplopia or exophthalmos, and adequate mouth opening with no trismus. No comorbidities were present.

**Figure 1:**
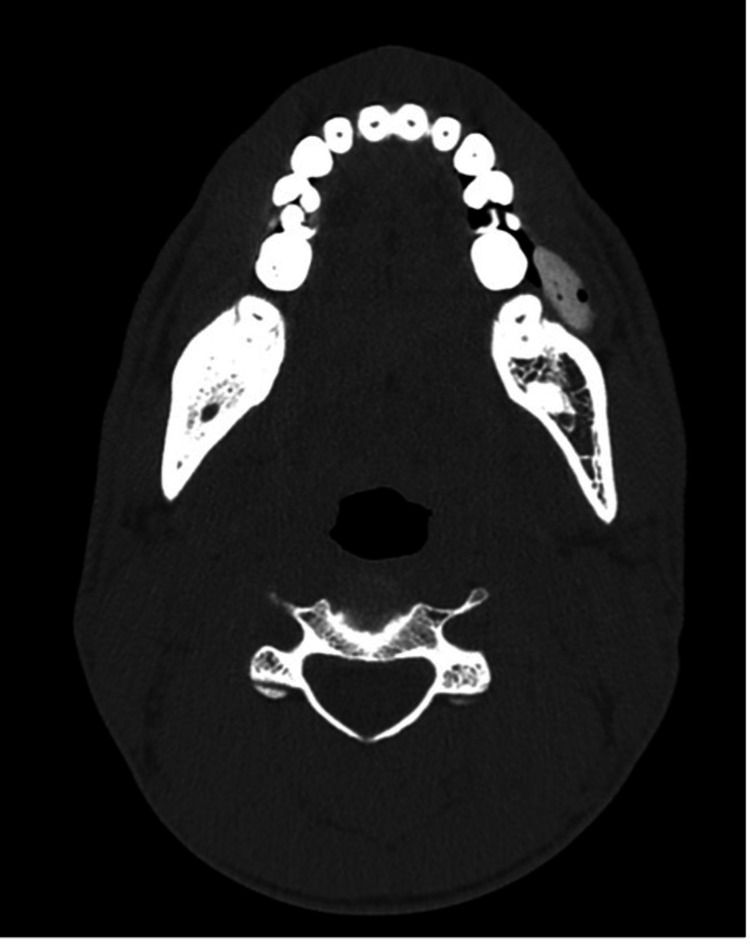
Axial image in the bone window showing a circumscribed, ovoid, hyperdense mass containing a few air locules in the inferior left buccal space adjacent to the left lower molar teeth.

**Figure 2:**
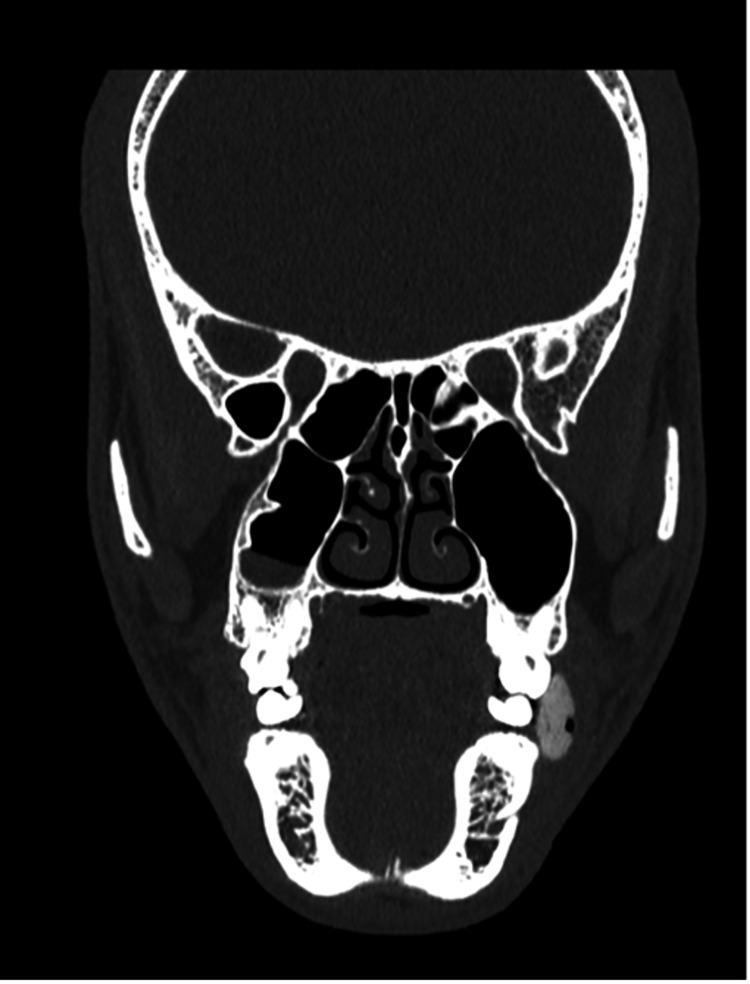
Coronal CT image in the bone window showing a circumscribed, ovoid, hyperdense mass containing a few air locules in the inferior left buccal space adjacent to the left lower molar teeth. CT: computed tomography

**Figure 3:**
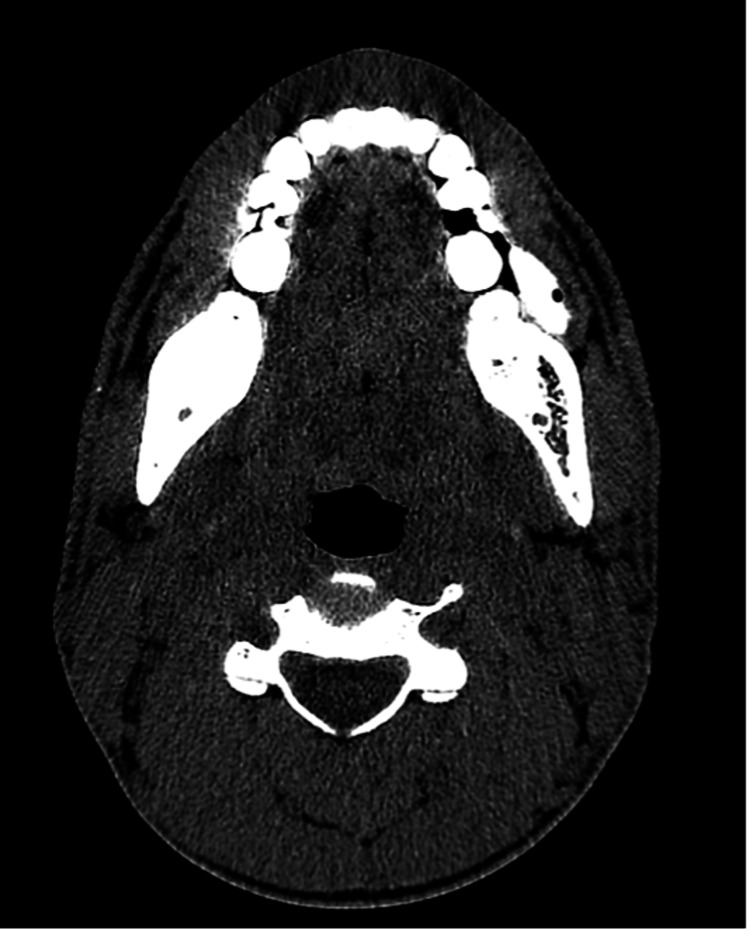
Axial CT non-contrast image in the soft tissue window showing a circumscribed, ovoid, hyperdense mass with internal gas locule in the inferior left buccal space adjacent to the left lower molar teeth. CT: computed tomography

**Figure 4:**
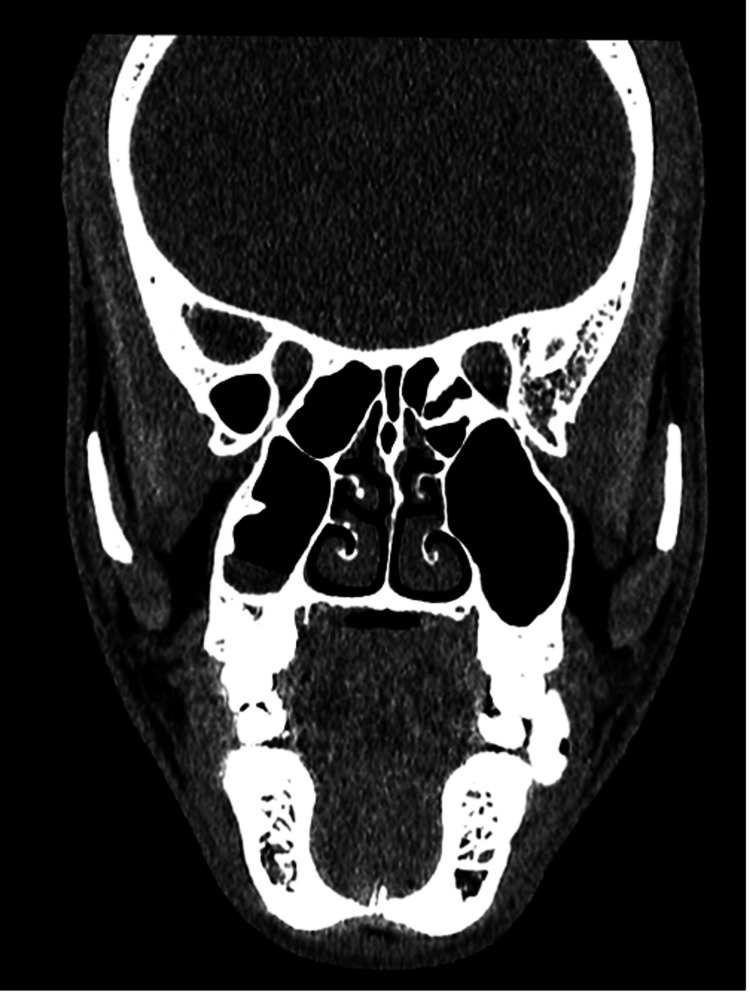
Coronal CT non-contrast image in the soft tissue window showing a circumscribed, ovoid, hyperdense mass with internal gas locule in the inferior left buccal space adjacent to the left lower molar teeth. CT: computed tomography

**Figure 5:**
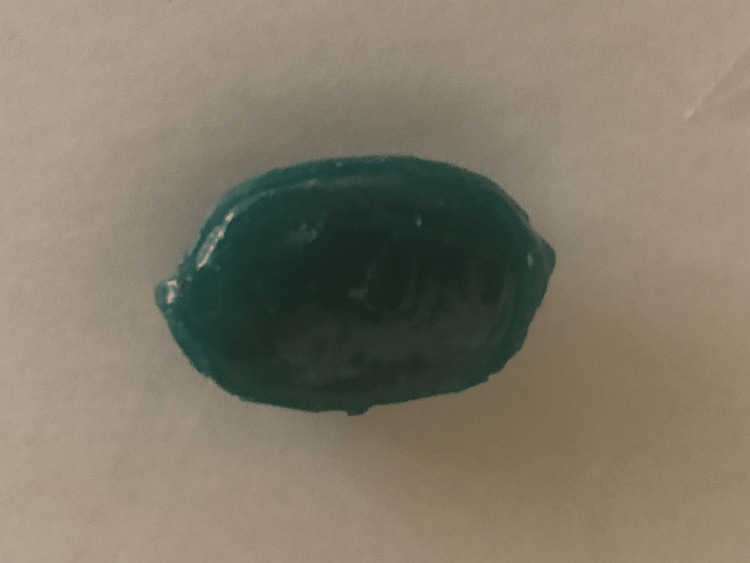
Photograph demonstrating the IOFB (hard candy). IOFB: intraoral foreign body

A facial bone CT scan revealed multiple facial and orbital fractures and a 1.8 cm (anterior-posterior) × 0.7 cm (transverse) circumscribed, ovoid, hyperdensity, just lateral to the lower molars in the inferior left buccal space (Figures 1-4). Internal pockets of air were noted centrally within this mass. Its mean Hounsfield unit (HU) was approximately 534 and ranged from 332 HU to 670 HU, being inconsistent with the densities of soft tissue, bone, metal, or blood. It was subsequently diagnosed as an IOFB based on imaging features and conversation with the patient. The IOFB in this case turned out to be a hard candy known as a “Diana PowerMint” (Figure 5).

## Discussion

An understanding of the imaging features of comestible IOFBs is essential for distinguishing these from true intraoral pathology, as many smaller and softer IOFBs can be easily confused with pathology [2]. Common comestible IOFBs, such as hard candies, chewing gum, soft candies, and tobacco, possess a diverse range of distinguishable radiological features. In the setting of trauma, as encountered in our case, the importance of differentiation between IOFBs, such as glass, metal, or wood from comestible IOFBs such as those above, is highlighted [3]. While instructing patients to remove comestible IOFBs prior to imaging remains a top priority of radiographers/technicians, it is sometimes unavoidably missed.

The hard candy in our case was circumscribed and ovoid in shape, with attenuation values ranging from 332 to 670 HU and internal air locules. Chewing gum, on the other hand, demonstrates a hyperdense appearance in comparison to soft tissues with Hounsfield units (HU) ranging from 100 to 400 depending on their composition. This IOFB tends to be found in mucosal recesses or adjacent to teeth attached to the gingiva or hard palate [4]. Soft chewable candies lack a characteristic shape, making them more difficult to appreciate on a scan. They are of lower average radiodensity and amorphous shape and are usually held against the teeth or located in mucosal recesses. Chewing tobacco has a heterogenous texture with mixed tissue and air attenuation with a rim of denser material, usually located in the gingivobuccal sulcus [2].

It is essential to recognize comestible IOFBs and differentiate them from traumatic foreign bodies on cross-sectional imaging. Metal objects such as bullets or nails usually have a much higher density (HU greater than 3,000), with the presence of streak artifacts. Glass has a polygonal shape and has high attenuation values, with HU ranging from 500 to 900. Wooden foreign bodies, such as splinters, usually are cylindrical in shape with a long axis and small diameter and have variable attenuation values, making them somewhat difficult to distinguish [5].

Comestible IOFBs may also resemble other pathological lesions. Dental abscesses occur in much of the same locations but are more heterogenous, poorly circumscribed, and usually contain low attenuation fluid within. Buccal mucosal squamous cell carcinoma also occurs in these locations but is usually lobulated, poorly circumscribed, heterogenous, and enhanced with multiple hypodense areas suggestive of collections or necrotic components.

These imaging features contrast significantly with the hard candy IOFB in our case, as it is relatively well-circumscribed and homogenous compared to abscesses and neoplasms, has much lower attenuation values than metals, and is differently shaped to wooden and glass foreign bodies.

It is of paramount importance that comestible intraoral foreign bodies be removed prior to imaging, but this can prove to be a challenge in situations where a patient has restricted mouth opening, is unconscious, or is unable to vocalize. Direct confirmation of these foreign bodies is difficult in these circumstances. Thus, knowledge of their imaging features plays an important role in their recognition. Furthermore, when these lesions are identified in their typical locations in the oral cavity, they should be regarded as benign and incidental [3].

## Conclusions

In conclusion, it is important to identify comestible IOFBs and differentiate them from true pathology to avoid unwarranted patient distress and further imaging or procedures that are unnecessary and costly.
